# Economic contributions of pharmaceutical interventions by pharmacists: a retrospective report in Japan

**DOI:** 10.1186/s40545-016-0073-7

**Published:** 2016-07-19

**Authors:** Daiki Yasunaga, Yuichi Tasaka, Satoshi Murakami, Akihiro Tanaka, Mamoru Tanaka, Hiroaki Araki

**Affiliations:** Division of Pharmacy, Ehime University Hospital, Shitsukawa, Toon, Ehime 791-0295 Japan

## Abstract

**Background:**

Pharmacists in Japan currently play a key role in patient hospital care. Their responsibilities include filling prescriptions, checking a patient’s medication history, and providing appropriate information to other health care workers. More importantly, pharmacists’ interventions can also result in reductions in adverse drug reactions (ADRs) and, ultimately, in cost savings. This study aimed to determine the economic value of such interventions at a hospital in Japan.

**Methods:**

At a single Japanese hospital, we analyzed 1452 pharmaceutical interventions by pharmacists, including recommending antibiotic dosage regimens, attending ward rounds with multidisciplinary health providers, providing drug information, and reporting ADRs. We classified the interventions into 13 categories. Using data from the PreAVOID Report by the Japanese Society of Hospital Pharmacists, along with previous studies, we estimated the cost savings of the interventions.

**Results:**

Various savings could be realized through appropriate interventions by hospital pharmacists. Based on the amount paid by the Pharmaceuticals and Medical Devices Agency, we calculated the cost savings associated with preventing serious ADRs as 21,400 USD ($) per case. The cost savings for recommendations related to transvenous antimicrobial therapy amounted to $1900 per patient. Pharmacists’ interventions were able to prevent 12 cases of serious ADRs.

**Conclusions:**

Determining the economic value of pharmacists’ interventions is an important means of appraising the current role of hospital pharmacists. Our evaluation demonstrates the positive economic effects of pharmacists’ interventions in a hospital setting.

## Background

Pharmacists today play a greater role in providing pharmacotherapeutics to patients [1]. However, to date, the economic contribution of various pharmaceutical interventions in a medical setting has not been thoroughly investigated in Japan. In this study, we calculated the economic impact of pharmaceutical interventions, including multidisciplinary teamwork, using an evidence-based approach.

It has been reported that in the United States of America, 6.7 % of the adverse effects resulting from pharmaceuticals administered to hospitalized patients are considered serious, and 0.32 % are fatal [2]. Similar figures have also appeared in a recent review [3]. Another US study has shown that patients who experience adverse effects have longer hospital stays and higher mortality rates than those who do not [4]. In addition, some studies have demonstrated that medical costs from pharmaceutical adverse effects are increasing annually [5, 6]. In 2011 in Japan, 959 cases of adverse drug reactions (ADRs) were referred to the ADR relief services of the Pharmaceuticals and Medical Devices Agency (PMDA). Those ADRs cost the PMDA $20,583,890 [7]. In Japan, the PMDA provides a medical allowance for harm to health that results from incorrect use of drugs (e.g., diseases and disabilities requiring hospitalization caused by adverse effects of drugs at hospitals and clinics).

The most important role of a pharmacist is to ensure effective, safe drug therapy for the patient. In preventing drug-related adverse events, the hospital pharmacist acts as a risk manager for hospitalized patients. Furthermore, the pharmaceutical evidence-based interventions of pharmacists play a useful role in medical treatment [8, 9]. A ward-based pharmaceutical service was introduced as a medical treatment bonus after fiscal 2012 in Japan; since then, the role of the pharmacist in hospital wards has expanded. Pharmacists have a number of responsibilities on the ward, including filling prescriptions, monitoring patients’ drug histories, avoiding drug interactions, providing drug information to medical staff, and recommending drug regimens. Pharmacists now also perform a pharmaceutical service for outpatients receiving chemotherapy. It has been reported that $565,664 per year could be saved by avoiding serious ADRs through continuous pharmaceutical interventions by pharmacists in US emergency care [10]. In addition, Niwa reported that a savings of 301,290,758 JPY ($3,012,907) per year per patient could be achieved by establishing a program to ensure the appropriate use of antimicrobial drugs in Japanese hospitals [11]. To date, however, no study has examined the economic impact of individual pharmaceutical interventions by pharmacists in Japan; therefore, this was the goal of the present study.

## Methods

The study was carried out in accordance with the guidelines for the care for human study adopted by the Ethics Committee of Ehime University Hospital (Ehime, Japan; approval number 1408004 of the review board). This investigation focuses on a single hospital and is a retrospective report of pharmacy interventions at Ehime University Hospital. Therefore, we did not obtain written informed consent for study participation. As of March 2014, the hospital had 626 beds and 41 pharmacists. We analyzed pharmacy interventions, including recommending antibiotic dosage regimens, attending ward rounds with multidisciplinary health providers, providing drug information, and reporting ADRs. We examined a total of 1452 pharmaceutical interventions performed at Ehime University Hospital from April 2013 to March 2014. We recorded and stored interventions in a web-based severity reaction database built by the Japanese Society of Hospital Pharmacists (JSHP) [12]. We categorized the 1452 reports into 13 types, following the classification used in Hamblin et al. [10]: (1) avoidance of serious ADRs; (2) transvenous antimicrobial therapy interventions; (3) switch from intravenous to oral administration; (4) interventions concerning cancer chemotherapy; (5) avoidance of drug interactions; (6) renal dosing recommendations; (7) intravenous drug compatibility; (8) confirmation of medication history (presurgical cessation of antiplatelet drugs); (9) drug therapy consultation or recommendations; (10) monitoring recommendations; (11) ward rounds, multidisciplinary teamwork; (12) drug information; and (13) ADRs reported to the PMDA.

In view of the cost of ADRs to the PMDA, we estimated that there could be an average saving of $21,400 through the avoidance of serious ADRs in Japan: $20,583,890 (1 USD = 100 JPY)/959 = $21,464). In this study, we defined serious ADRs as serious adverse effects described in the printed information inserts in the drug packaging. We determined the cost savings with recommendations for transvenous antimicrobial therapy—the dosage regimen of anti-methicillin-resistant *Staphylococcus aureus* (MRSA) drugs—with reference to the study by Niwa [11]. That report stated that daily savings of $272.37 per patient could be realized using the recommendations for transvenous antimicrobial therapy. At the hospital in the present study, anti-MRSA drugs are administered for an average of 7 days. Therefore, approximately $1900 per patient could be saved by appropriate pharmaceutical interventions ($272.37 × 7 = $1906.59). With regard to the switch from intravenous to oral administration, we calculated the cost difference per day multiplied by the number of days of oral administration.

Hamblin et al. reported that 2.6–5.21 % of pharmaceutical interventions, e.g., avoidance of drug interactions and attention to renal dosing recommendations, lead to the avoidance of serious ADRs [10]. We defined the economic contributions of individual pharmaceutical interventions based on Hamblin et al. and described the rate at which routine interventions could prevent serious ADRs (2.6–5.21 %). Adverse effects can occur with any drug, but such effects are particularly common with anticancer agents, and these drugs also represent the highest risk from the standpoint of reaction severity. Therefore, according to the risk of expression of ADRs, we classified previously described pharmaceutical interventions (4) to (9) into three categories: interventions for cancer chemotherapy, high-risk drugs exclude anticancer agents, and others. We defined the economic contribution for each pharmaceutical intervention. For interventions related to cancer chemotherapy, the contribution was approximately $1120 ($21,400 × 5.21 % = $1118.27). The contribution for high-risk drugs was about $840 ($21,400 × 3.91 % [the intermediate value between 2.6 % and 5.21 %] = $839.24). The contribution for others amounted to about $560 ($21,400 × 2.6 % = $558.06). In this study, we defined high-risk drugs according to JSHP business guidelines for these drugs [12]. Furthermore, based on the Hamblin et al. report [10], the medical economic contributions of pharmaceutical interventions (10) to (13) were defined as $0 (Table 1).

**Table 1:** Classification of pharmaceutical interventions and cost savings

Intervention type	Cost savings
1. Avoidance of serious ADRs	Benefits paid by PMDA to sufferers of ADRs in 2013: USD $20,583,890 Number of incidents: 959
	Average amount: $21,464 i.e., $21,400/case
2. Transvenous antimicrobial therapy interventions	$272.37/patient/day × 7 days^a^ = $1906.59/patient, i.e., $1900/patient
3. Switch from intravenous to oral administration	Difference in cost between intravenous and oral administration per day × days of oral administration
4. Interventions concerning cancer chemotherapy 5. Avoidance of drug interactions 6. Renal dosing recommendations 7. Intravenous drug compatibility 8. Confirmation of medication history (presurgical cessation of antiplatelet drugs) 9. Drug therapy consultation or recommendations	Likelihood that a general intervention leads to preventing an ADR ranges from 2.6 to 5.21 % Most risky drug therapy: cancer chemotherapy $21,464 × 5.21 % = $1118.27, i.e., $1120/case Intermediate risky drug therapy: high-risk drugs defined by JSHP $21,464 × 3.91 % = $839.24, i.e., $840/case Normal drug therapy: others $21,464 × 2.60 % = $558.06, i.e., $560/case
10. Monitoring recommendations 11. Ward rounds, multidisciplinary teamwork 12. Drug information 13. ADRs reported to PMDA	These types are not directly reflected in the cost estimation, i.e., $0

^a^The average number of days that anti-MRSA drugs were used at the study hospital

Multiple interventions for a single patient were counted as one intervention

*ADR* serious adverse drug reaction, *PMDA* Pharmaceuticals and Medical Devices Agency, *JSHP* Japanese Society of Hospital Pharmacists

## Results

Table 2 shows the results of the classified pharmaceutical interventions. We grouped the 1452 interventions into the 13 categories described above. There were 640 instances of drug information, e.g., information given to doctors, pharmacists, nurses, or other health professionals. We estimated the medical economic contribution from these pharmaceutical interventions to be $876,017.

**Table 2:** Estimation of annual economic impact

Intervention type			Number	Cost savings assigned per case (USD)	Total (USD)	Intervention class
1	Avoidance of serious ADRs		12	21,400	256,800	Quality/safety improved
2	Transvenous antimicrobial therapy interventions		172	1900	325,080	Pharmacotherapy improved
3	Switch from intravenous to oral administration	Voriconazole	55	165.1	9078	Cost saving
		Linezolid	32	96.9	3099	
4	Interventions concerning cancer chemotherapy		82	1120	91,840	Pharmacotherapy improved
5	Avoidance of drug interactions	High risk	2	840	1680	Pharmacotherapy improved
		Normal	56	560	31,360	
6	Renal dosing recommendations	High risk	7	840	5880	Pharmacotherapy improved
		Normal	43	560	24,080	
7	Intravenous drug compatibility	High risk	1	840	840	Pharmacotherapy improved
		Normal	2	560	1120	
8	Confirmation of medication history (presurgical cessation of antiplatelet drugs)	High risk	13	840	10,920	Quality/safety improved
		Normal	4	560	2240	
9	Drug therapy consultation or recommendations	High risk	34	840	285,60	Pharmacotherapy improved
		Normal	149	560	834,40	
10	Monitoring recommendations		19	0	0	Pharmacotherapy improved
11	Ward rounds, multidisciplinary teamwork	ICT	28	0	0	Quality/safety improved
		NST	45	0	0	
		PCT	52	0	0	
12	Drug information		640	0	0	Provider education
13	ADRs reported to PMDA		4	0	0	Quality/safety improved
		Total	1452	-	876,017	

*ADRs* serious adverse drug reactions, *ICT* infection control team, *NST* nutrition support team, *PCT* pain control team, *PMDA* Pharmaceutical and Medical Devices Agency

There were 12 instances of avoidance of serious ADRs, for which the savings were $256,800 (Table 3). Chemotherapy accounted for seven cases and avoidance of renal failure for two cases. There were 172 cases of transvenous antimicrobial therapy interventions, primarily involving Therapeutic Drug Monitoring: 121 of vancomycin (VCM); 37 of teicoplanin (TEIC); six of patients who switched to TEIC from VCM or vice versa; one of linezolid (LZD) one of arbekacin (ABK); and six of others. Total savings were $325,080. Regarding the change from intravenous to oral administration, voriconazole (VRCZ) featured twice and LZD four times. The administration period of the oral antimicrobial was 55 days for VRCZ and 32 days for LZD.

**Table 3:** Avoidance of serious adverse drug reactions

No.	Case	Pharmaceutical intervention
1	Renal function worsening due to a combination of fibrates and statins	Discontinue fibrates
2	Lithium intoxication and acute renal failure in patients orally administered lithium carbonate	Measure blood lithium concentration and discontinue lithium carbonate
3	Liver dysfunction because of phenytoin	Change to other anti-epileptic drugs
4	Hypoglycemia with oral diabetes drugs	Reduce dose of oral diabetes drugs
5	Pancytopenia after an increase in carbamazepine dosage	Discontinue carbamazepine
6	Bevacizumab administered to a patient with planned tooth extraction	Change to chemotherapy without bevacizumab
7	Start of chemotherapy for grade 4 neutropenic patients	Postpone chemotherapy
8	No blood test after chemotherapy (grade 4 neutropenia)	Recommend blood test
9	Onset of grade 2 peripheral neuropathy after chemotherapy	Begin adjuvant analgesics
10	Anaphylaxis by premedication at start of chemotherapy	Change premedication
11	Start of chemotherapy for patients untreated for HBV-DNA-positive conversion	Postpone chemotherapy and begin oral administration of entecavir
12	Start of chemotherapy for patients untreated for HBV-DNA detection	Begin oral administration of entecavir

*HBV* Hepatitis B virus

**Fig. 1:**
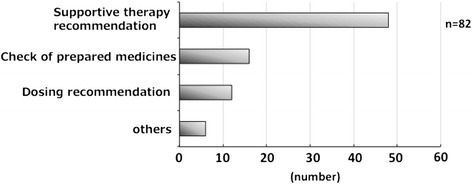
Interventions for cancer chemotherapy

Figure 1 shows the interventions for cancer chemotherapy. There were 48 interventions for supportive therapy recommendations, 16 for checking prepared drugs, 12 for dosing recommendations, and six other interventions, with total savings of $91,840. The interventions mainly involved suggestions of appropriate antiemetic drugs and avoidance of serious ADRs.

Table 4 presents the cases of avoidance of drug interactions, which represented savings of $33,040. Three interventions involved contraindications for coadministration, and 55 concerned a combination of issues. Most drug interactions were related to absorption inhibition.

**Table 4:** Avoidance of drug interactions

Contraindication for coadministration		Number
Azathioprine	Febuxostat	1
Atorvastatin	Bezafibrate	1
Ferrous citrate	Albumin tannate	1
Combination of issues		Number
Magnesium oxide	Cefdinir	13
Magnesium oxide	Oral new quinolone	13
Oral iron supplement	Cefdinir	9
Oral iron supplement	Levofloxacin	6
Oral iron supplement	Magnesium oxide	5
Antimicrobial	Lactomin	3
Pentazocine	Morphine	1
Famotidine	Itraconazole	1
Tacrolimus	Clarithromycin	1
Polystyrene sulfonate calcium	Magnesium oxide	1
Cefdinir	Sucralfate	1
SM powder	Levofloxacin	1
Total		58

*SM* Sankyo Magen Mittel

Oral iron supplement: ferrous citrate, ferrous fumarate, soluble ferric pyrophosphate

Antimicrobial: ampicillin/sulbactam, levofloxacin, erythromycin

Oral new quinolone: levofloxacin, minomycin, ciprofloxacin

There were 50 cases of renal dosing recommendations: for the main drugs famotidine, allopurinol, and levofloxacin, there were 12, 8, and six interventions, respectively, with combined savings of $29,960. Pharmacists were able to contribute to preventing renal function aggravation and various adverse effects, such as hypoglycemia, by decreasing the dosage of oral antidiabetic medication and changing to an agent other than loxoprofen sodium for patients with reduced renal function.

There were three cases involving intravenous drug compatibility, for the drugs heparin and fat emulsion, iron oxide and normal saline, and VCM and micafungin; the savings were $1960.

**Fig. 2:**
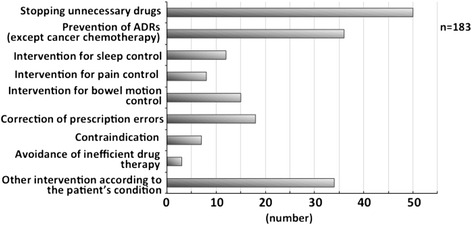
Drug therapy consultations or recommendations

Regarding drug therapy consultation or recommendations, there were 50 cases of discontinuing unnecessary drugs, 36 for the prevention of ADRs (except cancer chemotherapy), and 12 for sleep control, with savings of $112,000. A further eight interventions were recorded for pain control, 15 for bowel motion control, and 18 for the correction of prescription errors. There were seven cases of contraindication, three for avoiding inefficient drug therapy, and 34 for other interventions according to the patient’s condition (Fig. 2). Active pharmaceutical interventions depended on the state of the patient; for example, pain control and bowel motion control were commonly implemented interventions.

**Fig. 3:**
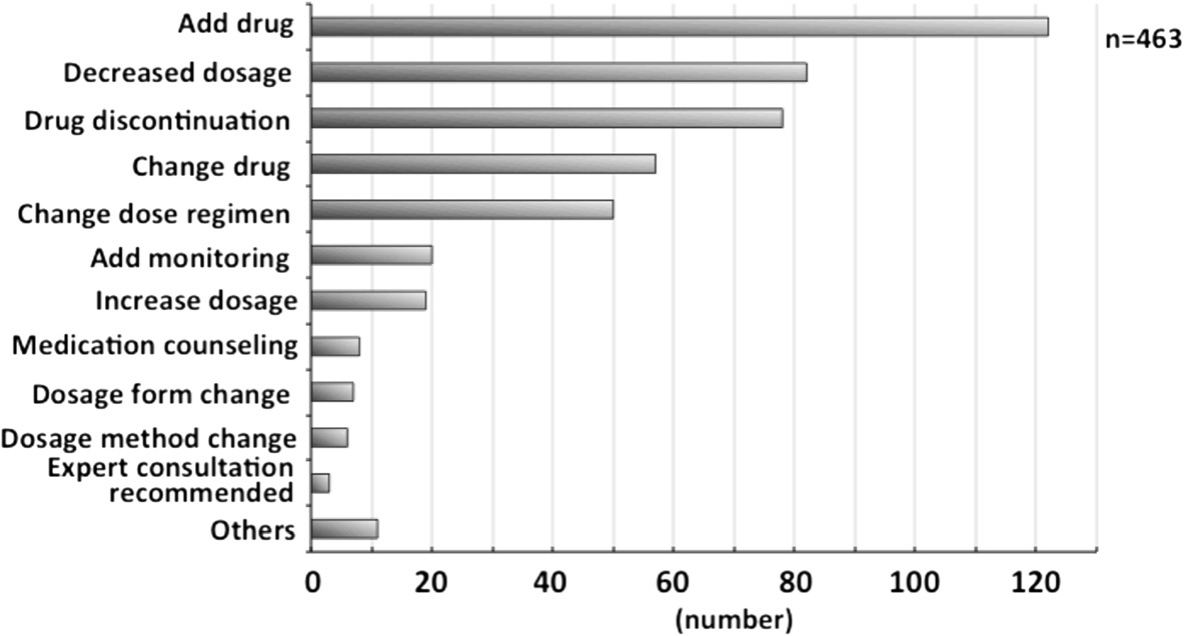
Method of intervention

In all, 463 interventions concerned additional drugs. In 122 (26.3 %) cases, the dosage was decreased; there were 82 cases (17.7 %) of drug discontinuation and 78 cases (16.8 %) of the drugs’ being changed; 57 (12.3 %) cases represented a change in the dose regimen and 50 cases (10.8 %) other interventions (Fig. 3). All the interventions regarding monitoring recommendations included the recommendation for hepatitis B screening.

## Discussion

One study has reported that pharmaceutical inquiries regarding prescriptions by community pharmacists were effective from a medical economic perspective, representing savings of $11,888 [13]. In the present investigation, we conducted an economic evaluation of the effect of individual pharmaceutical interventions by clinical pharmacists. It is generally considered necessary to calculate costs induced by the adverse effects of drugs. However, it is very difficult to determine such costs, due to the great variety of drugs and their adverse effects. In this study, the individual outcomes in the absence of interventions were unknown. Therefore, we could not assess the expected economic contribution when an ADRs occurred because medical expenses vary greatly.

The damage relief system for ADRs offered by Japan’s PMDA involves compensation contingent on appropriate use of the pharmaceuticals. We calculated the average compensation paid to a patient in Japan after a serious adverse effect at $21,400, which is $6000 higher than the figure reported by Hamblin et al. [10] One reason for the high cost of PMDA compensation is that it includes a patient benefit (The PMDA is an incorporated administrative agency under the Ministry of Health, Labour and Welfare, and we therefore considered it appropriate to include that benefit when calculating the economic contributions).

Our analysis of 1452 pharmaceutical interventions indicated that serious ADRs were avoided in 12 cases. This underlines the importance of the pharmacist in ward drug duties to ensure the safe management of patient medical treatment. In transvenous antimicrobial therapy interventions, most cases involved VCM, TEIC, and ABK. Approximately 70 % of interventions concerned VCM cases. VCM is recommended as a first-line drug in MRSA infectious disease treatment guidelines in Japan. Switch therapy constitutes a change in administration of antimicrobial drugs from an intravenous drip to oral medication. It has been shown that switch therapy can reduce both medical expenses and length of hospital stay if introduced at an early stage of treatment [14]. From a cost perspective and because of their high bioavailability, we considered VRCZ and LZD to be suitable for switch therapy. With switch therapy, we estimated the cost savings at approximately $12,000 due to cost differences in the drugs.

The most common intervention for cancer chemotherapy involved recommendations for supportive therapy. Therefore, we believe that pharmacists can prevent or relieve the frequent onset of serious adverse effects of an anticancer agent by suggesting supportive therapy. Cancer chemotherapy often causes critical adverse effects. Accordingly, we assumed the rate of serious ADRs to be 5.21 %.

Contraindications for coadministration were extracted by the computer system and printed on prescriptions, so such risks were basically averted in our hospital. Because cases that involve only a prescription do not qualify as interventions in this study, only three cases of actual interventions occurred in this category.

Intravenous drug incompatibility not only reduces the titer of the drug, but also may cause a side effect. Because various infusions may be mixed in this route of administration, a careful chemical judgment is required. Only three cases occurred in this study, but ensuring compatibility is an important pharmaceutical intervention.

Regarding the confirmation of medication history, pharmacists commonly intervened in the discontinuation of preoperative anticoagulants and their postoperative readministration. For example, with angiotensin II receptor antagonists and angiotensin-converting enzyme inhibitors, it is recommended that the pharmacist cancel such prescriptions before an operation under practice guidelines for surgical medical care [15]. Similarly, many other drugs and anticoagulants should be discontinued before an operation. We would argue that such pharmacist interventions lead to the avoidance of adverse events during an operation and indicate a pharmacist’s appropriate assessment of drug use in hospitals.

Pharmacists need to target their efforts particularly toward improving medication safety to prevent ADRs. Patients who experience an ADR require a longer hospital stay, which results in greater hospital costs [5, 16]. Any intervention that reduces ADRs will have a significant impact on patient care and health care costs. Drug costs can be directly increased or decreased by adding or removing a drug prescription. However, since the present study focuses on the economic contribution related to the prevention of adverse effect, we have not included the costs associated with adding or removing drugs.

There are four intervention types (Monitoring recommendations, Ward rounds, multidisciplinary teamwork, Drug information, ADRs reported to PMDA) for which we did not estimate economic effects in this study. Cooperation among medical care team members is known to be a strong contributor to symptom relaxation and improvement of the patient’s QOL [17]. Regarding ward rounds and multidisciplinary teamwork, there were 28 cases of infection control team, 45 cases of nutrition support team, and 52 cases of pain control team. The intervention category of drug information, including education of ward staff, contained 640 cases. These interventions therefore offer many opportunities for economic contributions.

Lazarou et al. [2] found that 50 % of the adverse effects of pharmaceutical products were preventable [2]. Bond and Raehl [4] estimated that $30 billion a year could be saved by avoiding adverse effects—even if such effects were preventable in only approximately 50 % of hospitals in the USA [4] (just a small number of hospitals are registered with the US adverse effect-reporting system). In Japan, the proportion of medical expenses attributed to ADRs is unknown. The present study is the first to investigate medical-related economic contributions in Japan, and we therefore believe that it contributes to the avoidance of adverse effects.

## Conclusions

In this study, we conducted medical-related economic evaluations using an original estimation system. We calculated the effects of individual pharmaceutical interventions by pharmacists on reducing medical costs. In this way, we were able to determine the economic contributions of such interventions. Other pharmacists can use this work to evaluate interventions economically, using our classification and estimation system.

## Abbreviations

ADRs, adverse drug reactions; LZD, linezolid; MRSA, anti-methicillin-resistant *Staphylococcus aureus*; PMDA, Pharmaceuticals and Medical Devices Agency; VCM, vancomycin; VRCZ, voriconazole
